# Silicon carbide-free graphene growth on silicon for lithium-ion battery with high volumetric energy density

**DOI:** 10.1038/ncomms8393

**Published:** 2015-06-25

**Authors:** In Hyuk Son, Jong Hwan Park, Soonchul Kwon, Seongyong Park, Mark H. Rümmeli, Alicja Bachmatiuk, Hyun Jae Song, Junhwan Ku, Jang Wook Choi, Jae-man Choi, Seok-Gwang Doo, Hyuk Chang

**Affiliations:** 1 Energy Material Lab, Material Research Center, Samsung Advanced Institute of Technology, Samsung Electronics Co., Ltd, 130 Samsung-ro, Yeongtong-gu, Suwon-si, Gyeonggi-do 443-803, Republic of Korea; 2 Analytical Engineering Group, Platform Technology Lab, Samsung Advanced Institute of Technology, Samsung Electronics Co., Ltd, 130 Samsung-ro, Yeongtong-gu, Suwon-si, Gyeonggi-do 443-803, Republic of Korea; 3 IBS Center for Integrated Nanostructure Physics, Institute for Basic Science (IBS), Daejon 305-701, Republic of Korea; 4 Department of Energy Science (DoES), Department of Physics, Sungkyunkwan University, Suwon 440-746, Republic of Korea; 5 Centre of Polymer and Carbon Materials, Polish Academy of Sciences, M. Curie-Sklodowskiej 34, Zabrze 41-819, Poland; 6 IFW Dresden, Institute for Complex materials, PO Box D-01171, Dresden 270116, Germany; 7 Nano Electronics Lab, Device and System Research Center, Samsung Advanced Institute of Technology, Samsung Electronics Co., Ltd, 130 Samsung-ro, Yeongtong-gu, Suwon-si, Gyeonggi-do 443-803, Republic of Korea; 8 Graduate School of Energy, Environment, Water, and Sustainability (EEWS), Korea Advanced Institute of Science and Technology (KAIST), Daejeon 305-701, Republic of Korea; 9 Material Research Center, Samsung Advanced Institute of Technology, Samsung Electronics Co., Ltd, 130 Samsung-ro, Yeongtong-gu, Suwon-si, Gyeonggi-do 443-803, Republic of Korea

## Abstract

Silicon is receiving discernable attention as an active material for next generation lithium-ion battery anodes because of its unparalleled gravimetric capacity. However, the large volume change of silicon over charge–discharge cycles weakens its competitiveness in the volumetric energy density and cycle life. Here we report direct graphene growth over silicon nanoparticles without silicon carbide formation. The graphene layers anchored onto the silicon surface accommodate the volume expansion of silicon via a sliding process between adjacent graphene layers. When paired with a commercial lithium cobalt oxide cathode, the silicon carbide-free graphene coating allows the full cell to reach volumetric energy densities of 972 and 700 Wh l^−1^ at first and 200th cycle, respectively, 1.8 and 1.5 times higher than those of current commercial lithium-ion batteries. This observation suggests that two-dimensional layered structure of graphene and its silicon carbide-free integration with silicon can serve as a prototype in advancing silicon anodes to commercially viable technology.

## Abstract (web summary)

The volume expansion of silicon is a big problem in lithium-ion batteries with silicon anodes. Here, the authors report direct graphene growth on silicon nanoparticles, which effectively mitigates the problem, leading to excellent electrochemical performance.

## Graphical abstract

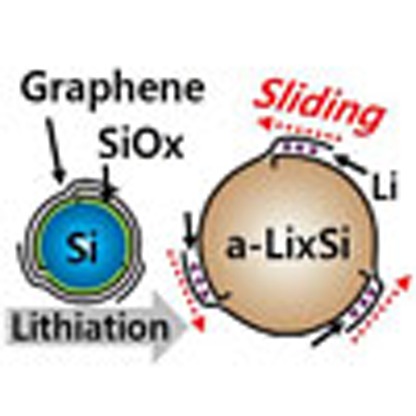

The theoretical gravimetric capacity of silicon (Si) reaches almost 4,000 mAh g^−1^. This unparalleled value has stimulated the battery community to invest considerable research efforts because the high gravimetric capacity enables one to increase the energy densities of lithium-ion batteries (LIBs) significantly, and thus bring future LIB applications, such as electrical vehicles, to a reality^1^,^2^,^3^,^4^,^5^. In the past decade, diverse advanced electrode structures^6^,^7^,^8^,^9^,^10^,^11^,^12^,^13^,^14^,^15^,^16^ and binder designs^17^,^18^,^19^ were developed to resolve chronic capacity fading issues originating from the large volume change of Si, leading to substantially improved cycling performance even over thousands of cycles^11^. In spite of the promising gravimetric value and substantial progress in cycle life, most of Si anodes demonstrated to date have focused primarily on the gravimetric capacity but have not offered a similar promise in their volumetric capacity because existing electrode designs rely on pre-defined void space to accommodate the volume expansion of Si. In many LIB applications including portable electronics, however, the volumetric energy density is a critical parameter in determining battery performance. Together with a relatively inferior cycle life, weak volumetric energy density is presently a major bottleneck in implementing Si anodes in commercial cells. To meet this critical demand, Si anode technology needs to be revisited with different electrode designs that offer stable cycling performance while the electrode volume is minimized.

Although a variety of Si morphologies and their composites with other conductive materials are currently available, for an immediate solution to actual manufacturing that requires high-standard quality control, an easy and scalable synthesis of active Si components is crucial. From a practical application standpoint, among a vast number of viable candidates, the use of commercial Si nanoparticles (NPs) with a simple and efficient conductive surface coating would be highly desirable. Various conductive materials including amorphous carbon (AC) have been investigated as coating materials for Si NP anodes^8^,^20^,^21^. However, most of them fail to deliver stable long-term cycling performance because the implemented coating materials are unable to accommodate the volume expansion of Si and consequently fracture over repeated cycles.

In an attempt to address the limitation of previous conductive coatings as well as to achieve good cycling performance with a significantly higher volumetric energy density, in this study, we adopt multilayer graphene directly grown on the Si surface as a coating material. The two-dimensional (2D) layered character of graphene provides a unique and efficient operation of Si anodes since multilayered graphene can accommodate Si volume expansion via a sliding process between adjacent layers without the need to provide void space *a priori* in the as-made electrode. Also, the graphene-coated Si NP pellet exhibits 12.8 S cm^−1^ at a marginal graphene content of 1 wt% through a high percolation network. To this end, we overcome a challenge of growing graphene directly on Si surfaces without Si carbide (SiC) formation by developing a chemical vapour deposition (CVD) process that involves a mild oxidant. SiC formation is fatal in Si anode operations because SiC is an electrical insulator with poor defect characteristics. Moreover, SiC is inactive in reacting with Li ions and consequently hinders Li ion diffusion into the Si phase. With the assistance of the graphene interlayer sliding process and enhanced conductivity, the graphene-coated Si NPs reach a volumetric capacity of 2,500 mAh cm^−3^ (versus 550 mAh cm^−3^ of commercial graphite), the highest value among those reported to date for any LIB anodes while exhibiting excellent cycling and rate performance.

## Results

### SiC-free graphene growth on Si

**Figure 1:**
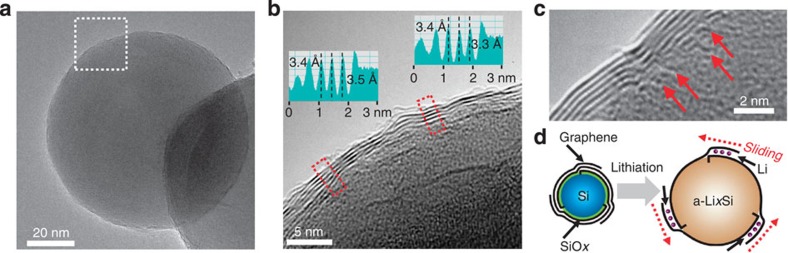
SiC-free graphene growth on Si NPs. (**a**) A low-magnification TEM image of Gr–Si NP. (**b**) A higher-magnification TEM image for the same Gr–Si NP from the white box in **a**. (Insets) The line profiles from the two red boxes indicate that the interlayer spacing between graphene layers is ∼3.4 Å, in good agreement with that of typical graphene layers based on van der Waals interaction. (**c**) A high-magnification TEM image visualizing the origins (red arrows) from which individual graphene layers grow. (**d**) A schematic illustration showing the sliding process of the graphene coating layers that can buffer the volume expansion of Si.

The direct growth of high-quality graphene on Si via CVD process has proven challenging^22^ because typical graphene synthesis conditions require a reducing atmosphere that tends to strip the native Si oxide layer off the Si surface, and then drives a reaction between Si and decomposed carbon precursors to form SiC^23^. Our initial approach of using methane (CH_4_) as a carbon precursor mixed with H_2_ was indeed unable to achieve a graphene growth on the Si NPs (Supplementary Fig. 1), thereby yielding only β-SiC (Supplementary Fig. 2). To overcome this limitation, we included carbon dioxide (CO_2_), a mild oxidant, in the CVD process along with CH_4_ (refs 24, 25). The inclusion of CO_2_ allows one to avoid the formation of SiC and also lower the growth temperature compared with well-known graphene growth on surface^23^. In our experiment, at 900 °C, the graphene growth was incomplete or inhomogeneous (Supplementary Fig. 3), whereas graphene growth at 1,100 °C produced oxide layers on the Si surface that are too thick for efficient Li ion diffusion (Supplementary Fig. 4). At an intermediate temperature of 1,000 °C, 2–10 layers of graphene were formed as shown in transmission electron microscope (TEM) images (Fig. 1a,b) clearly displaying the layered structure as well as the interlayer distance near 3.4 Å (Fig. 1b, inset). Closer inspection indicates that individual layers are anchored directly to the Si particle surface at their ends (red arrows in Fig. 1c) and lie parallel to the Si surface. These well-aligned graphene layers can maintain their layered stacking structure even during lithiation via a sliding process (Fig. 1d), thus providing an elegant means to accommodate the volume expansion of Si. Graphene growth has been attempted on Si dioxide (SiO_2_) surfaces using CH_4_ and H_2_ (refs 26, 27). However, the graphene growth in those processes was very inefficient or does not avoid SiC formation. The inefficient growth is ascribed to insufficient catalytic sites on SiO_2_ surface. By contrast, in our process, the CO_2_ addition generates more catalytic sites in the form of SiO_*x*_ with some defects.

**Figure 2:**
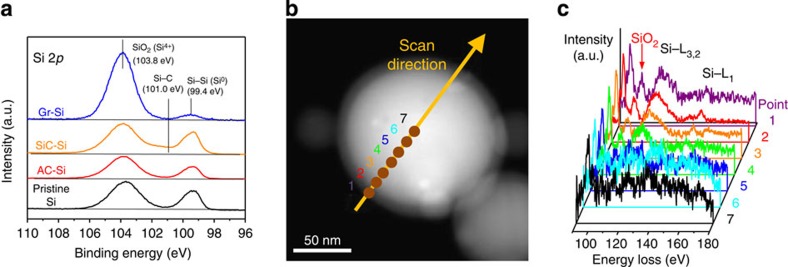
Verification of SiC-free growth. (**a**) X-ray photoelectron spectroscopy spectra in Si 2*p* band for Gr–Si, SiC–Si, AC–Si and pristine Si. (**b**) A scanning TEM image of a Gr–Si NP. (**c**) EELS spectra for the spots denoted in **b**.

The SiC-free graphene growth was verified by both bulk scale and single-particle scale analyses. X-ray photoelectron spectroscopy profiles in Si 2*p* band (Fig. 2a) as well as X-ray diffraction (XRD) spectra (Supplementary Fig. 2) show no peaks corresponding to SiC for graphene-coated Si (Gr–Si), AC-coated Si (AC–Si) and pristine Si, in contrast to a control (SiC–Si) sample synthesized through a CO_2_-free route using CH_4_ and H_2_. On the other hand, a scanning TEM imaging using high-angle annular dark field showed that the Si NP have a core–shell structure with brighter core and thin relatively darker shell attributed to a Si core and an oxide coating (Fig. 2b). Electron energy loss spectroscopy (EELS) spectra (Fig. 2c) obtained for multiple spots across the NP did not exhibit any signals^28^ reflective of SiC formation, confirming SiC-free growth in the current growth process. Also, SiO_2_ signals at 108 eV were detected at points 1 and 2 more strongly than the other spots in the centre, implying that the SiO_2_ surface serves as catalytic sites for graphene growth. The persistent presence of the SiO_2_ surface layers was also verified by XRD (Supplementary Fig. 2) and EELS (Fig. 2b,c) characterization.

### *In situ* TEM analysis

**Figure 3:**
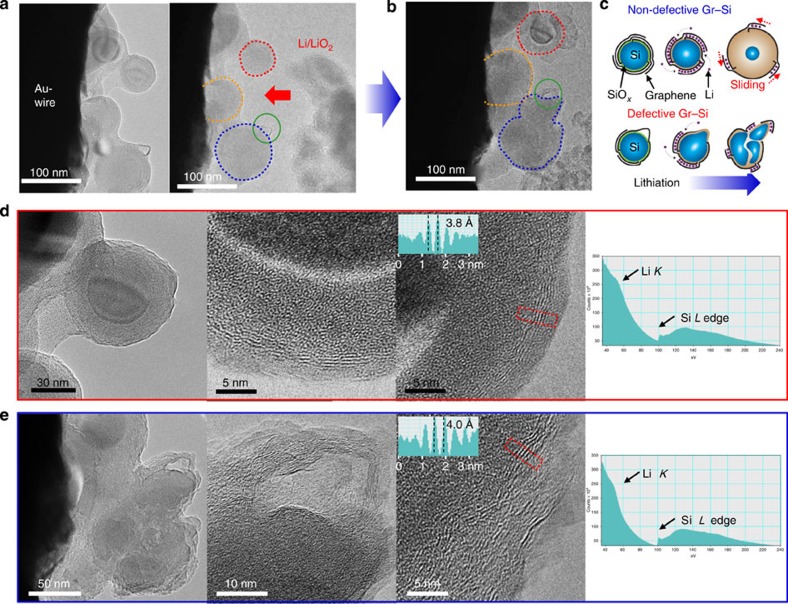
***In situ*** **TEM analysis.** (**a**) Gr–Si NPs attached to the surface of Au wire and a second Li/LiO_2_ electrode. (**b**) The same Gr–Si NPs after lithiation. (**c**) A schematic summary of lithiated Gr–Si NPs for both non-defective and defective graphene encapsulation. (**d**,**e**) Close up TEM images for (**d**) non-defective particle (the one circled with the red line in **a** and **b**) and (**e**) defective particle (the one circled with the blue line in **a** and **b**). The EELS spectra in both cases confirm the lithiation. The line profiles from the red boxes in both cases show increased interlayer distances of ca. 3.8 Å, reflective of lithiation into the graphene interlayer space.

We now turn to the use of Gr–Si as an anode material in LIBs. The lithiation of the Gr–Si NPs (1 wt% graphene) was monitored in real time during their volume expansion using *in situ* TEM analysis^29^,^30^,^31^,^32^. In the actual experiment, some Gr–Si particles were placed onto a gold (Au)-fixed electrode. A second, but movable, electrode with Li/LiO_2_ at its tip was positioned to just come into contact with the Gr–Si NPs to lithiate them (Fig. 3a). Once contact with the second electrode was made, the particles began to swell (Fig. 3b). Greater details on this process can be seen in Supplementary Fig. 5 and Supplementary Movies 1–3. Two types of expanding structures were observed, namely non-defective particles and defective particles as highlighted in Fig. 3b,c. In cases where the graphene fully encapsulates the particle and has no obvious defects (Fig. 3d), the diameter increased by ∼30% (220% volume expansion). At the end of lithiation, the interlayer distance of the graphene layers increased to 3.8 Å reflective of Li intercalation^33^, and the layered characteristic of the graphitic coating was preserved all round the NP (Fig. 3d), suggesting a sliding process between layers as illustrated in Fig. 3c. In cases where a defective region exists (see green circle in Fig. 3a), upon swelling, the inner particle pulverizes and ruptures through the defect (Fig. 3e). The observed distinct fracture behaviours are unlikely to be from particle size, as an encapsulated particle bigger than the fractured one in Fig. 3 did not fracture (see the indicated particles at 0 and 40 s in Supplementary Fig. 5). Also, recent observation^34^ indicates that if not encapsulated, even smaller Si particles (<50 nm) can fracture due to Li concentration gradient within the particles that can create a substantial stress. In fact, the critical size of Si particle fracture is case dependent, and is related on how efficiently the stress built up during volume expansion is able to be released. The critical size can extend to >200 nm (ref. 35).

Local EELS analysis during lithiation verifies the lithiation of Si for both cases through a blurred Li *K*-edge near 55 eV as well as Si *L*-edge at 100 eV (Fig. 3d,e; ref. 36). The layer shown on the surface of graphene in Fig. 3d is attributed to Li/Li_2_O residue. In essence, the graphene layers preserve the integration with Si through their chemically bonded roots on the Si surface, and at the same time, the sliding process allows the few layer graphene coating to accommodate the volume expansion of Si. In a separate *ex situ* TEM experiment with the Gr–Si LIB electrode, it was observed that the average number of graphene layers measured at different locations decreased from 9.4 to 5.6 layers (Supplementary Fig. 6) after the first lithiation, reconfirming the interlayer sliding process.

The graphene encapsulation and interlayer sliding process exert a clamping force that helps to maintain Si particle integrity during volume expansion. However, this clamping mechanism does not appear to affect Li diffusion, as good specific capacities are achieved in the electrochemical measurements. It is also notable that the volume expansion of particles was, on the whole, uniform in all radial directions even though the contact point with the second electrode is highly localized. This highlights the graphene layers' propensity for homogeneous Li ion diffusion into the Si core (see red and orange dotted lines in Fig. 3a,b). The *in situ* analysis also indicates that Li diffusion through the graphene layers is pretty fast, in agreement with previous studies^30^,^37^. Also, it is noted that the observed clamping effect is mainly from the graphene coating layers rather than surface native oxide layers because the surface oxide layers are too thin to have a significant effect. The clamping effect based on surface oxide layers has been observed only when Si has hollow structures^7^,^11^ or the oxide layers are very thick^38^.

### Conductivity and electrochemical measurements

**Figure 4:**
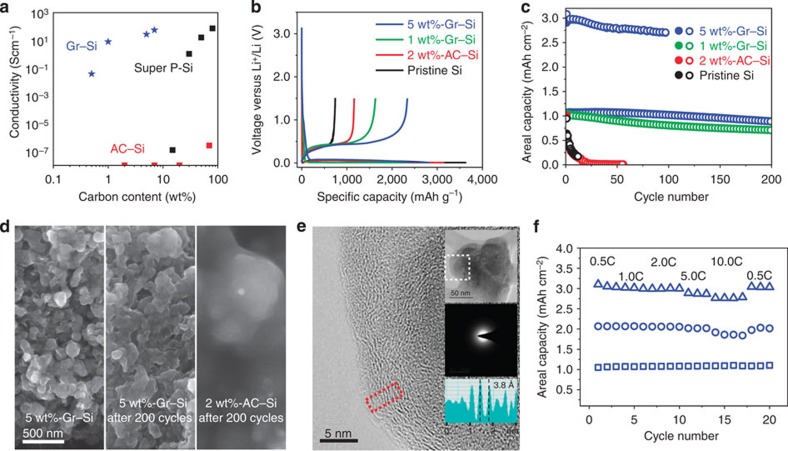
Conductivity and electrochemical analysis. (**a**) The pellet conductivities of Gr–Si, AC–Si and Super P–Si at different carbon contents. (**b**) The first lithiation–delithiation profiles. The electrode thicknesses of 5 wt%-Gr–Si, 1 wt%-Gr–Si, 2 wt%-AC–Si and pristine Si (Super P 1 wt%) are 4.5, 5.0, 8.3 and 12.3 μm, respectively. (**c**) The lithiation (filled circle) and delithiation (open circle) capacity retentions of the same four samples. The potential was swept within a voltage window between 0.01 and 1.5 V at 0.5C. (**d**) The top-viewed SEM images: the 5 wt%-Gr–Si electrode (left) before and (middle) after 200 cycles and (right) the 2 wt%-AC–Si electrode after 200 cycles. (**e**) A high-magnification TEM image of 5 wt%-Gr–Si after 200 cycles obtained from the white box in the low-magnification TEM image (top inset). Middle inset: selected area electron diffraction showing amorphous nature of Si after cycling. Bottom inset: a line profile from the graphene layers in the red box displaying an interlayer distance of 3.8 Å. (**f**) Rate capability of 5 wt%-Gr–Si with different initial areal capacities (3.0, 2.0 and 1.0 mAh cm^−2^).

Upon graphene growth, the conductivity of the active material assembly becomes markedly enhanced. When pelletized in powder, pristine Si exhibited a low conductivity of <10^−7^ S cm^−1^, whereas even 1 wt% of graphene addition increased the conductivity remarkably to 12.8 S cm^−1^ (Fig. 4a). This enhancement is ascribed to an excellent percolation behaviour of the graphene coating layers in a way that the 2D graphene and its well-aligned layered stacking promote a well-connected conductive network (Supplementary Fig. 7), and is also in line with previous graphene–polymer composites^39^. The conductivity increased further to 38.3 S cm^−1^ for a graphene content of 5 wt%.

The electrochemical performance of the Gr–Si NPs was examined by preparing coin-type half-cells in which Li metal serves as the counter/reference electrode. For comparison, in addition to Gr–Si with different graphene contents, AC–Si and pristine Si were also investigated. The graphene content was controlled by the growth time while the growth temperature was fixed to 1,000 °C. All samples exhibited characteristic lithiation and delithiation plateaus at 0.1 and 0.4 V versus Li/Li^+^, respectively, in the first cycle (Fig. 4b; 0.05C, 1C=2,000 mA g^−1^, 0.01–1.5 V versus Li/Li^+^). 5 wt%-Gr–Si, 1 wt%-Gr–Si, 2 wt%-AC–Si and pristine Si exhibited lithiation/delithiation capacities of 2,820/2,340, 2,341/1,629, 3,143/1,160 and 3,632/741 mAh g^−1^, respectively, when Si and coated graphene are taken into account, leading to initial Coulombic efficiencies (CEs) of 83%, 69%, 37% and 20%. The low reversible capacity and initial CEs of AC–Si are attributed to the defects of the AC layers that trap Li ions.

The graphene coating makes a drastic difference in the cycling performance (Fig. 4c and Supplementary Fig. 8). For a fair comparison among the samples, we first set the areal capacity to 1 mAh cm^−2^. Under this constraint, the capacity fading of both 2 wt%-AC–Si and pristine Si was significant even for early cycles, indicating that a simple AC coating is not operational at all. Between both Gr–Si samples, 5 wt%-Gr–Si exhibited better performance than that of 1 wt%-Gr–Si (85% versus 70% retention after 200 cycles), suggesting that depending on the areal capacity, the graphene content becomes critical. The lower retention of 1 wt%-Gr–Si might be explained by occasional defect formation as observed in our *in situ* TEM investigation based on the same graphene content (Fig. 3e). For the 5 wt% graphene sample, the testing was expanded to even higher areal capacities up to 6 mAh cm^−2^ (Supplementary Fig. 8b). For areal capacities of 1, 3 and 6 mAh cm^−2^, the capacity retentions after 100 cycles were 96%, 91% and 43%, respectively. These samples also delivered good CEs of 99.6, 99.3, and 99.4% after 100 cycles, respectively. The effect of the conformal graphene coating on the structural integrity of Si NPs was elucidated by post-mortem scanning electron microscope (SEM) and TEM images after 200 cycles. Even after 200 cycles, the SEM image of 5 wt%-Gr–Si visualized individual NPs with clear particle boundaries (Fig. 4d). By contrast, the SEM image of 2 wt%-AC–Si showed that solid electrolyte interphase (SEI) layers overgrew and buried individual NPs, indicating the importance of the graphene coating on the stability of SEI layers. The TEM analyses (Fig. 4e and Supplementary Figs 9–11) of 5 wt%-Gr–Si visualized the preserved individual layers of the graphene coating, confirming their robust integration with Si NPs during the repeated large volume change again through the sliding process. In addition, the film thickness of 5 wt%-Gr–Si with 3 mAh cm^−2^ increased by 51.3% (15.0→22.7 μm) after the first cycle, which is substantially smaller than that (77.4%, 10.2→18.1 μm) of 5 wt%-Super P-pristine Si even with half the areal specific capacity of 1.5 mAh cm^−2^.

According to TEM images after the first delithiation (Supplementary Fig. 10) and the 200th delithiation (Supplementary Fig. 11), the interlayer distance of the graphene layers remained at ∼3.8 Å, indicating that the sliding process is not fully reversible. These phenomena are attributed to a combined effect from SEI layer formation and residual Li ions between the graphene layers, together with the smaller volume shrinkage during the first delithiation than the volume expansion of the first lithiation^40^,^41^. Nonetheless, the graphene layers remain rooted on the Si surfaces, continuously fulfilling their original roles as a conducting agent and a buffering medium to accommodate volume expansion of Si.

The graphene coating also has a marked effect on the rate capability utilizing its well-developed percolating network. Even at a high areal capacity at 3 mAh cm^−2^, a 20 times C-rate increase from 0.5 to 10 C still retained 90% of the initial capacity. It should be emphasized that the direct graphene growth on Si NPs used here is fundamentally different from previous works in which a simple mixture of Si NPs and reduced graphene oxide is prepared via a solution process in that in the case of the solution processes, complete encapsulation of Si NPs is not feasible^42^,^43^,^44^,^45^,^46^,^47^. To compensate this shortcoming, the reduced graphene oxide content needs to be increased significantly, which, in turn, sacrifices the specific capacity and CE. For similar reasons, none of previous coatings made of carbon nanomaterials prepared via solution processes has achieved stable cycling performance at areal capacities comparable to those in the present study.

**Figure 5:**
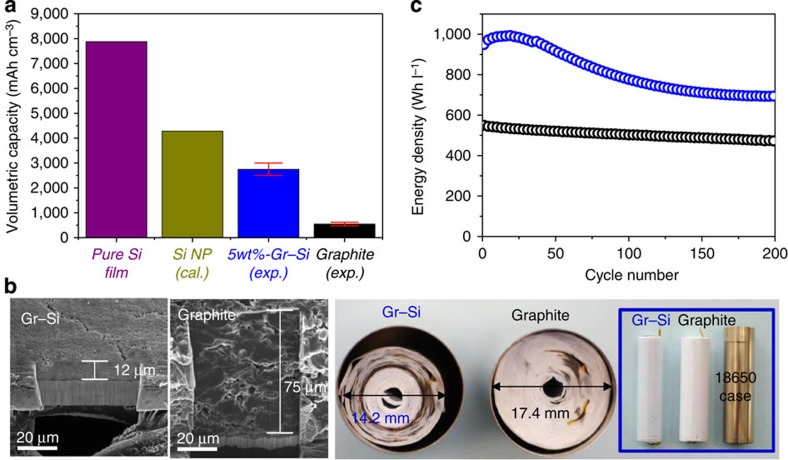
The volumetric energy density of 5 wt%-Gr–Si. (**a**) The volumetric capacities of pure Si film (calculation, cal.), theoretically packed Si NP film (calculation), 5 wt%-Si–Gr electrode (experimental) and graphite electrode (experimental). The value of theoretically packed Si NP film (calculation) was obtained by consideration of the gravimetric theoretical capacity of Si at room temperature (3,580 mAh g^−1^), the density of Si (2.2 g cm^−3^), the void portion in the theoretical particle packing (body centred, 0.32) and the binder content (∼20 wt%). (**b**) Cross-sectional SEM images of the 5 wt%-Gr–Si and commercial graphite electrodes (left). Top (right) and front (blue inset box) views of the 5 wt%-Gr–Si//LiCoO_2_ and graphite//LiCoO_2_ full cells with the same total energy (9.0 Wh). Both cells were wound into 18650 cylindrical cases with an identical winding tension. (**c**) The cycling performance of the 5 wt%-Si–Gr//LiCoO_2_ and graphite//LiCoO_2_ full cells. The 5 wt%-Gr–Si electrode in **b** and **c** is the one with 3.0 mAh cm^−2^ shown in Fig. 4c.

The volumetric capacity of Gr–Si was elucidated in comparison with those of the theoretical and commercial cases (Fig. 5a). The volumetric capacity of 5 wt%-Gr–Si (for the electrode with 3.0 mAh cm^−2^ in Fig. 4c) ranges from 2,500 to 3,000 mAh cm^−3^, which is close to the theoretical value (4,284 mAh cm^−3^) of the pristine Si particles on the assumption of their ideal packing (details in figure caption). The present values are far higher than that (550 mAh cm^−3^) of the current commercial graphite-based anodes. To further assess the practical viability of 5 wt%-Gr–Si, full cell performance was examined by pairing with a commercial cathode, LiCoO_2_. By consideration of the average cell voltage of 3.5 V, the areal capacity of 3.0 mAh cm^−2^, and a total electrode thickness of 108 μm (electrode+separator+current collector, Gr–Si thickness=15 μm), the volumetric energy density of the present full cell reaches 972 Wh l^−1^. This value is remarkable since it is 1.8 times as large as that (550 Wh l^−1^) of widely used current commercial LIBs calculated based on the same metric( http://www.samsungsdi.com/lithium-ion-battery/overview).

To see the improvement in the volumetric energy density in a more tangible manner, both the 5 wt%-Gr–Si (108 μm thick) and graphite electrodes (168 μm thick) were packed into commercial 18650 cylindrical cells with all other conditions (cathode and separator) fixed. When wound into a roll using a commercial taper tension controller, the roll diameter of the 5 wt%-Gr–Si full cell was 14.2 mm, which is significantly shorter than that (17.4 mm) of the commercial graphite counterpart (Fig. 5b), providing direct evidence of the increased volumetric energy density by the direct graphene growth. The present full cell exhibited 972 Wh l^−1^ at its initial cycle, and this value decreased to 700 Wh l^−1^ after 200 cycles (Fig. 5c). It is also worth noting that the capacity after 200 cycles is still 1.5 times higher than that (471 Wh l^−1^) of the graphite-based control cell. These volumetric energy densities of the graphene-based full cells are converted to 242.0 and 169.6 Wh kg^−1^ at the first and 200th cycles, which are ∼22% and 0% times higher than those of the graphite-based control full cell, respectively. In addition, despite the high volumetric energy density due to the dense graphene-coated Si particle packing, safety is unlikely to be an issue because the volume change of the anode is <10% of the entire cell volume.

## Discussion

In conclusion, we have developed a CVD process for the direct growth of graphene layers over Si NPs without SiC formation by utilizing CO_2_ as a mild oxidant. The individual graphene layers are directly anchored to the Si surfaces and thus remain integrated with Si during large volume changes of Si over repeated lithiation–delithiation cycles. The robust growth of graphene layers uniformly around each particle as well as over a large number of particles is attributed to the addition of CO_2_ in the CVD process that generates catalytic sites on the particle surface. The current approach involving CO_2_ is expected to hold true for other oxide surfaces that suffer from similar inefficient graphene growth and carbide formation.

The layered structure of graphene allows interlayer sliding upon the volume expansion of Si as well as a highly conductive percolating network, resulting in an unprecedented volumetric energy density of an LIB full cell with decent cycle life. Overall, the unique 2D character of graphene and the atom-level engineering of its interface with Si to avoid unwanted SiC formation will allow Si anode technology to make a meaningful step towards its wide commercialization.

## Methods

### CVD process for SiC-free graphene growth

For direct growth of graphene over the surface of Si NPs (Gr–Si), pristine Si NPs (average diameter ∼100 nm, Nanostructured and Amorphous Materials, Inc.) were introduced into a fixed-bed vertical tube reactor made of quartz (inner diameter=9 mm; refs 48, 49). The test gas was flowed through a reactor-containing bed from the top at atmospheric pressure. The loading of Si NPs in each batch was 0.1 g. Initially, the system was heated to target temperatures (900, 1,000 and 1,100 °C) at a ramping rate of 23 °C min^−1^ while a gas mixture of 50 s.c.c.m. CH_4_ and 50 s.c.c.m. CO_2_ was flowed. The temperature was maintained for 10 and 20 min, which results in 1 and 5 wt% of carbon content, respectively. The system was then cooled to room temperature (25 °C) in the presence of the mixed gas flow. For the synthesis of SiC–Si, the system is first heated to 1,000 °C (23 °C min^−1^) while H_2_ gas flowed at a rate of 100 s.c.c.m. This process was maintained for 60 min to reduce the native oxide layer over Si NPs. Next, the gas mixture consisting of 50 s.c.c.m. CH_4_ and 50 s.c.c.m. H_2_ was introduced for 10 min. AC–Si was synthesized by a known pyrolysis process (400 °C, 30 min) using citric acid as a carbon precursor^50^.

### Battery preparation and analysis

For the preparation of the battery electrode, slurry was prepared by dispersing active material (Gr–Si/AC–Si) and Li-polyacrylic acid (1.1 M) in a weight ratio of 80:20 in deionized water. The bare Si electrode, a control sample, was prepared by the same procedure, but the electrode components were pristine Si NPs, Super P and 1.1 M Li-polyacrylic acid. The binder content was fixed to 20 wt%, while the contents of pristine Si NPs and Super P were varied from 65 to 79 wt% and from 15 to 1 wt%, respectively. The half-cell was then assembled by using lithium foil as both reference and counter electrodes. Polyethylene membrane (Celgard) and 1.1 M lithium hexafluorophosphate (LiPF_6_) in ethylene carbonate:diethyl carbonate:fluoroethylene carbonate=2:6:2=v:v:v were used as separator and electrolyte, respectively. The mass loading of the active material ranged from 0.3 to 2.5 mg. The electrochemical properties were characterized under the galvanostatic mode by scanning the potential between 0.01 and 1.5 V versus Li/Li^+^ at 0.5C except the first cycle at 0.05C. The full cell performance of Gr–Si was characterized by paring with a LiCoO_2_ cathode with a fixed areal capacity of 3.2 mAh cm^−2^. The N/P ratio, defined by total capacity ratio between anode and cathode, was chosen to be 1.0–1.1.

### Physical and chemical analysis

XRD patterns were obtained by using an X-ray diffractometer (D8 Advance, Bruker Inc., 40 kV, 40 mA) engaging nickel-filtered Cu Kα radiation (*λ*=1.54 Å). The scan rate and interval were 4° min^−1^ and 0.02°, respectively. The X-ray photoelectron spectroscopy measurements were performed by using Physical Electronics (Quantera II, ULVAC-PHI, Inc.) with an Al Kα source (1,486.7 eV). The carbon content on Si NPs was quantitatively analysed (Supplementary Fig. 12) by using a thermogravimetry analyser (METTLER TOLEDO TGA/DSC1). High-resolution TEM, diffraction pattern and EELS analyses were carried out using FEI Titan Cubed 60–300 equipped with double Cs correctors and Gatan Quantum 965. The samples were also visualized using field-emission SEM (Nova NanoSEM 450S, FEI) to monitor the morphology change. The electronic conductivity of the pellet was measured using the four-probe DC method after pelletizing active powder at 20 kN (MCP-PD511, Mitsubishi).

### *In situ* TEM analysis

The *in situ* TEM experiments were performed in Titan 200 by using an electrical probing TEM holder. The Gr–Si NP solution was dropped onto a TEM grid until several Gr–Si NPs were located at the end of an Au wire electrode. Li metal was scratched onto a cleaved Pt/Ir electrode to generate a Li/Li_2_O counter electrode. The entire procedure for the sample loading was carried out in a dry room (<55 °C dew point, <0.5 % relative humidity at 25 °C). The TEM holder was moved to the TEM instrument inside a sealing box, and the holder was exposed to air for <30 s before being loaded into the TEM chamber. The manipulation of the probe tip was precisely controlled by a piezo-electric motor in order to make physical contacts between Gr–Si NPs and Li metal counter electrode. After secure contact was made, a constant bias from −0.5 to −5 V was applied. The microstructure evolution during lithiation was recorded as a movie clip.

## Additional information

**How to cite this article**: Son, I.H. *et al.* Silicon carbide-free graphene growth on silicon for lithium-ion battery with high volumetric energy density. *Nat. Commun.* 6:7393 doi: 10.1038/ncomms8393 (2015).

## Supplementary Material

Supplementary Figures Supplementary Figures 1-12

Supplementary Movie 1 In-situ TEM movie showing lithiation of graphene coated silicon nanoparticles by contact with a Li/LiO2 counter electrode

Supplementary Movie 2 In-situ TEM movie showing continuous swelling of graphene coated silicon nanoparticles during lithiation

Supplementary Movie 3 In-situ TEM movie showing the Li/LiO2 counter electrode detached from swollen graphene coated silicon nanoparticles
