# Supplementary material for: Silicon carbide-free graphene growth on silicon for lithium-ion battery with high volumetric energy density
Source: Nat Commun. 2015 Jun 25;6:7393. doi: 10.1038/ncomms8393 (PMC4491181; doi:10.1038/ncomms8393)
Supplement: Supplementary Figures — 1-12 [file ncomms8393-s1.pdf]

Supplementary Figures

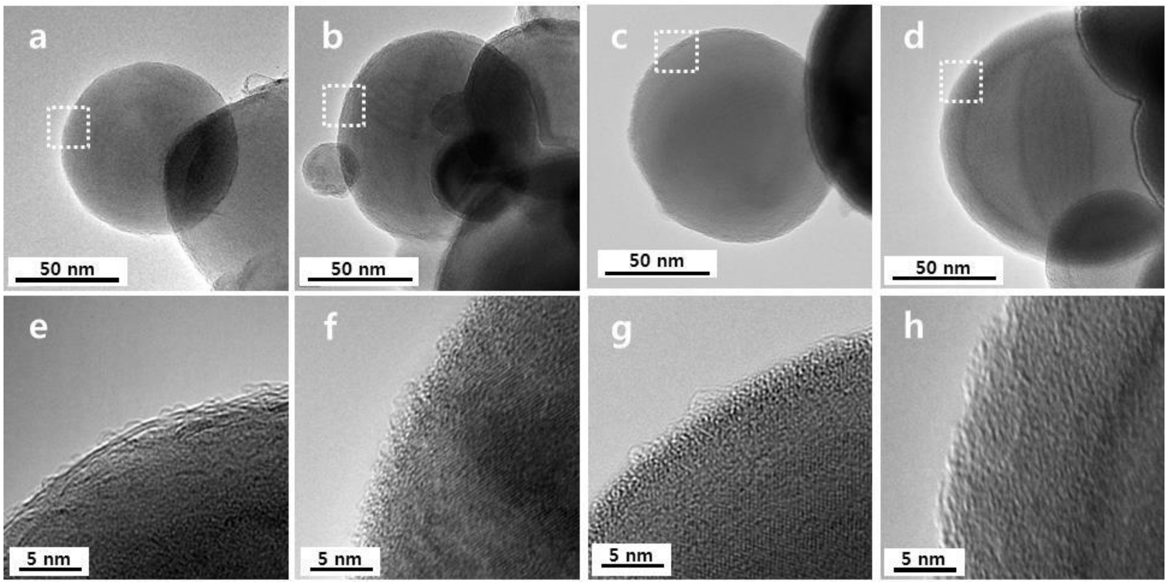

Supplementary Figure 1. TEM characterization of the Si active particles used in this study. Low and high magnification TEM images of (**a,e**) Gr-Si (CO_2 + CH_4, 1000 ^oC), (**b,f**) SiC-Si (H_2 + CH_4, 1000 ^oC), (**c,g**) AC-Si (citric acid, 400 ^oC), and (**d,h**) pristine Si.

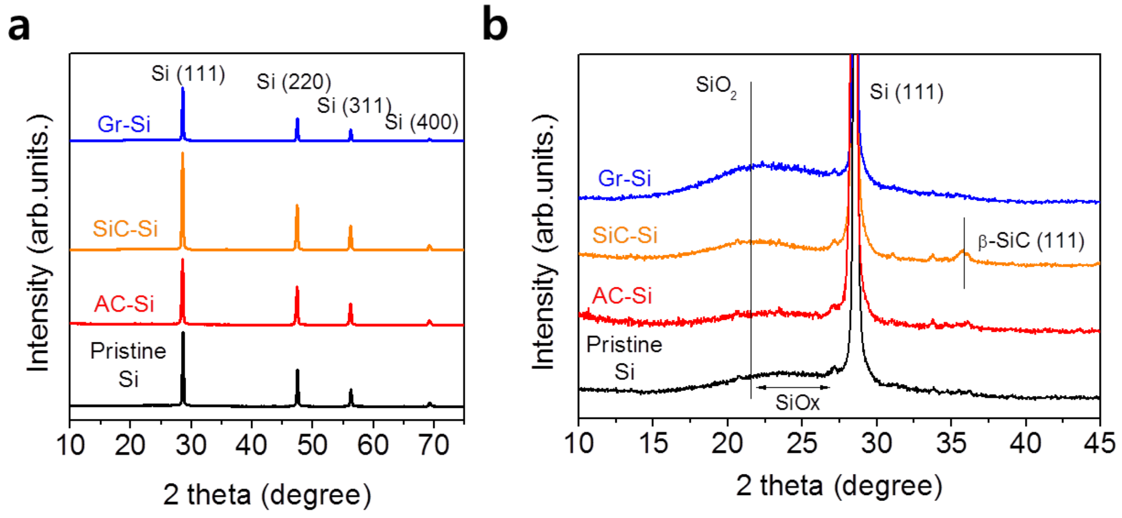

Supplementary Figure 2. XRD analysis of different Si NPs. XRD spectra of Gr-Si (synthesized at 1000 ^{o}C), SiC-Si, AC-Si, and pristine Si in (**a**) wide and (**b**) small 2 theta ranges. While SiC-Si shows a signal of β-SiC at 35.8°, Gr-Si exhibits no such peak confirming its SiC-free synthesis. Gr-Si also shows a broad peak around 22° indicating remaining SiO_2 surface layer.

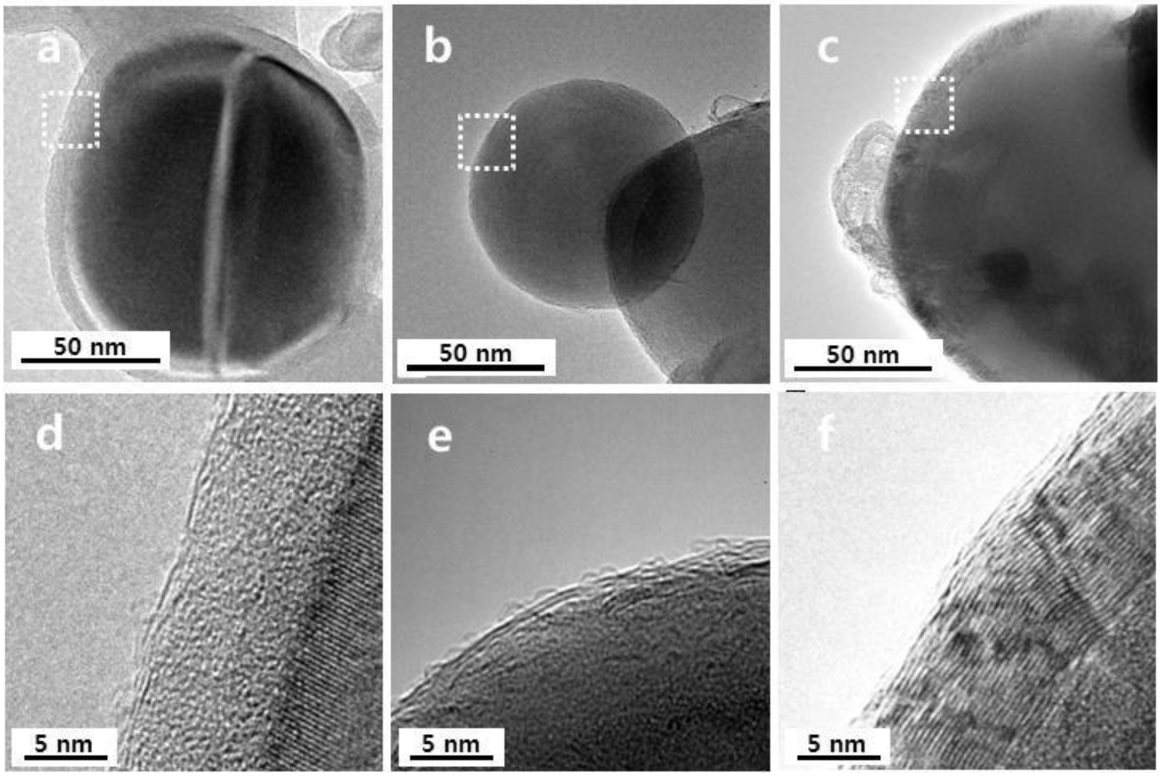

Supplementary Figure 3. TEM characterization of Gr-Si synthesized at different temperatures. Low and high magnification TEM images of Gr-Si synthesized at (**a,d**) 900 ^{o}C, (**b,e**) 1000 ^{o}C and (**c,f**) 1100 ^{o}C.

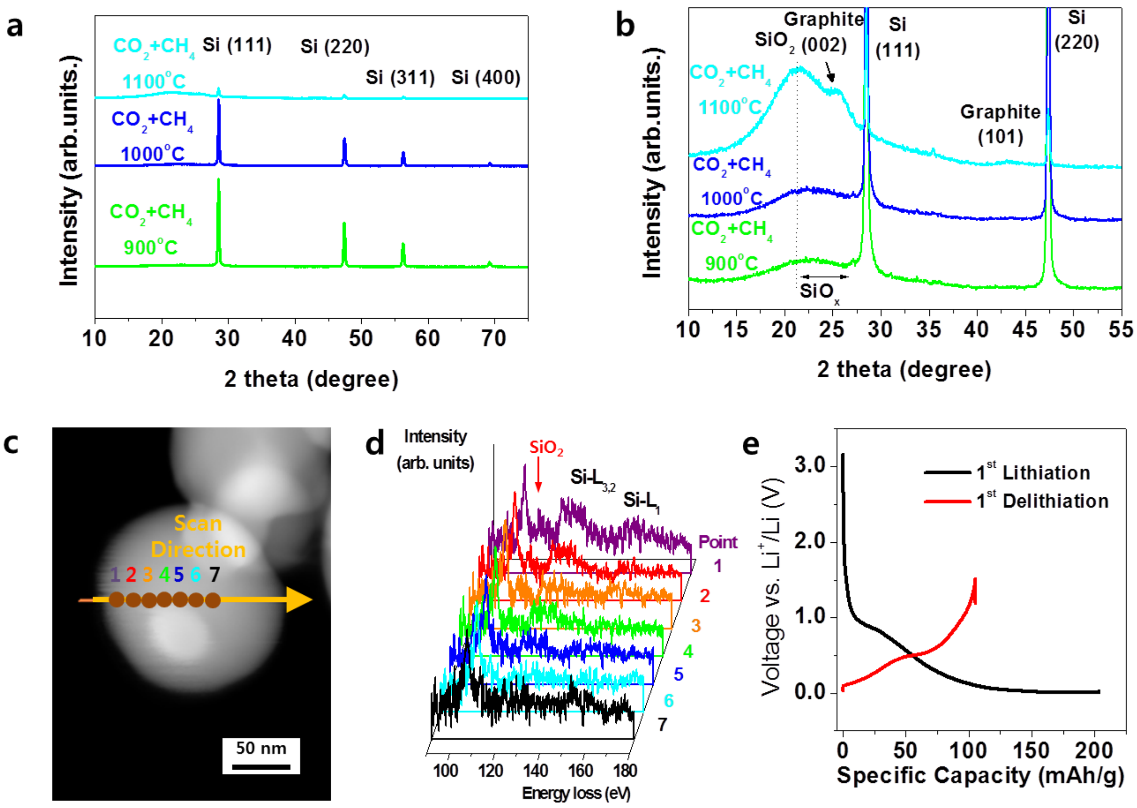

Supplementary Figure 4. Characterizations of Gr-Si synthesized at different temperatures. XRD spectra of Gr-Si NPs synthesized at 900, 1000 and 1100 °C in (**a**) wide and (**b**) small 2 theta ranges. (**c**) A STEM image of a Gr-Si NP synthesized at 1100 °C. (**d**) EELS spectra for the spots denoted in (**c**). (**e**) The first lithiation-delithiation profile of a half-cell based on Gr-Si synthesized at 1100 °C.

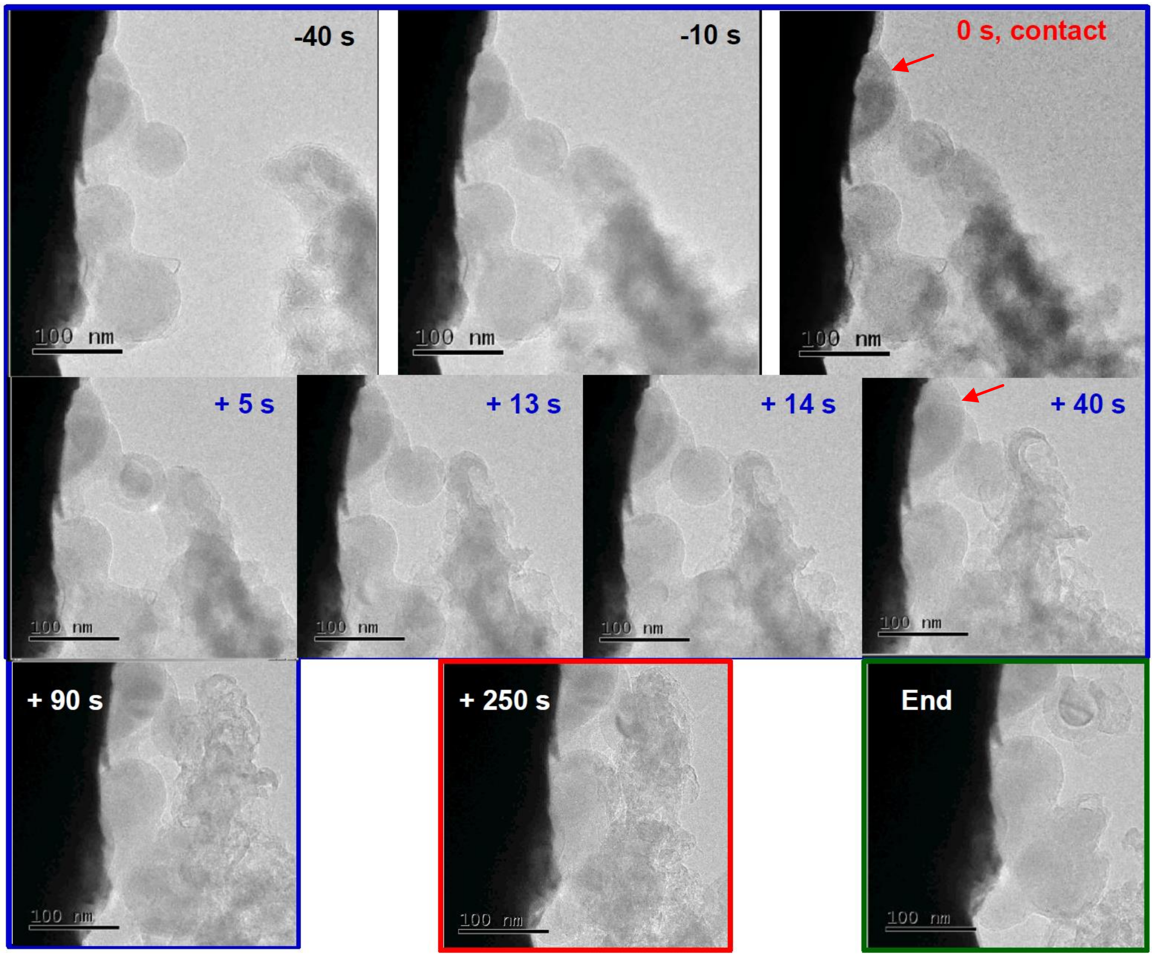

Supplementary Figure 5. *In-situ* TEM analysis. Screen capture images of Supplementary Movie 1 (blue line), Supplementary Movie 2 (red line), and Supplementary Movie 3 (green line).

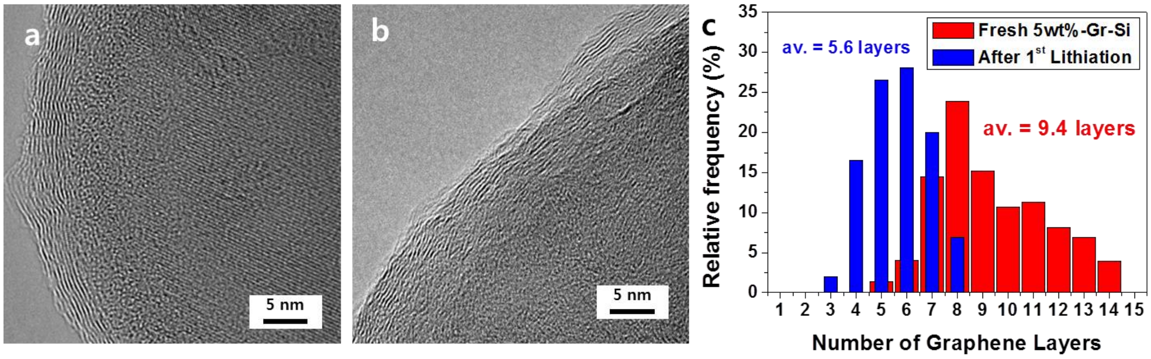

Supplementary Figure 6. *Ex-situ* TEM analysis of Gr-Si NPs before and after the 1st lithiation. Close up TEM images of 5wt%-Gr-Si NP (**a**) before and (**b**) after the 1st lithiation. (**c**) Statistics on the number of graphene layers on Si surface before and after the 1st lithiation.

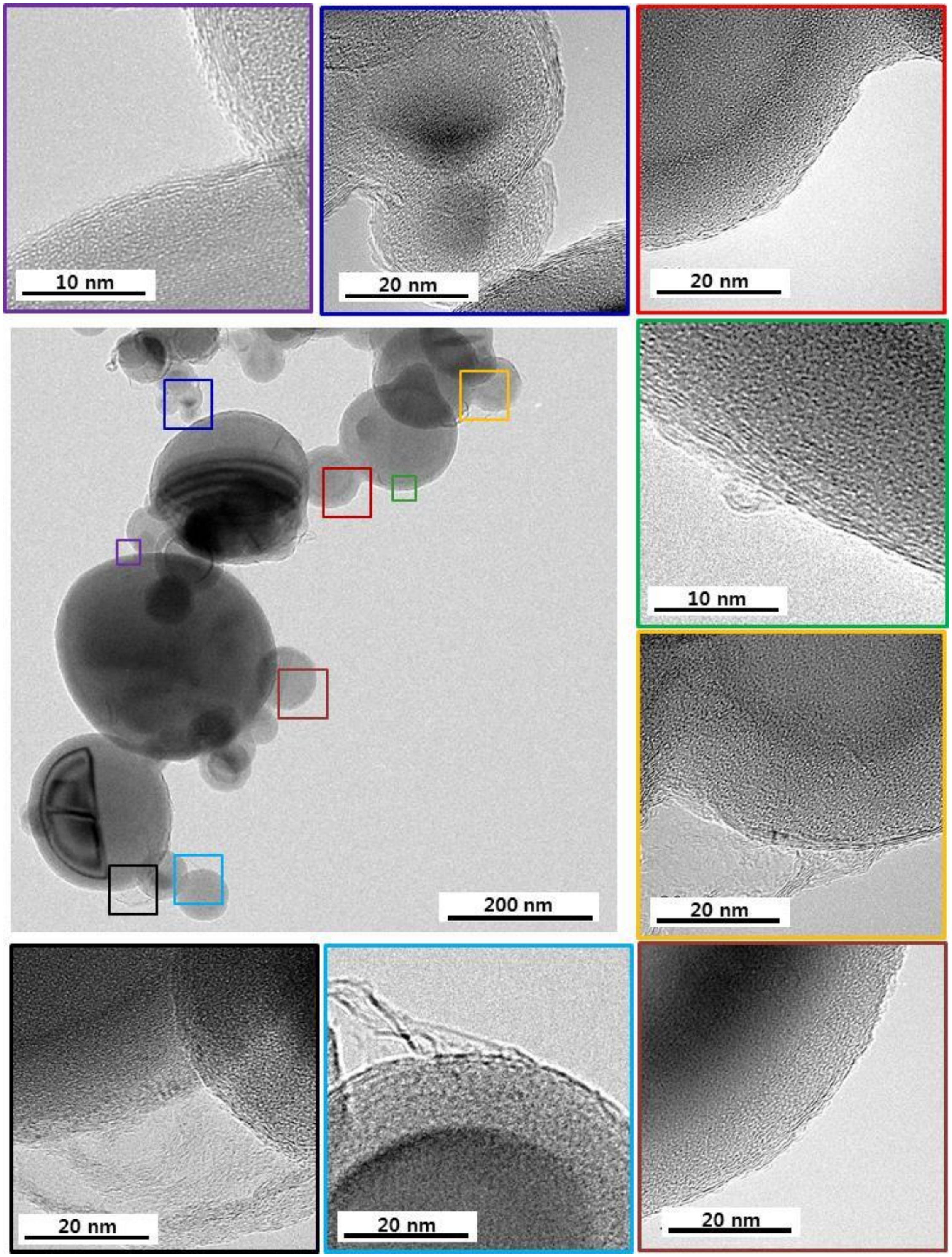

Supplementary Figure 7. Multi-spot TEM analysis of 1wt%-Gr-Si. A lower magnification TEM image of 1wt%-Gr-Si NP (CO_2+CH_4 at 1000 ^oC). Higher magnification HR-TEM mages from the color boxes of the center TEM image clearly show graphene layers on Si surface.

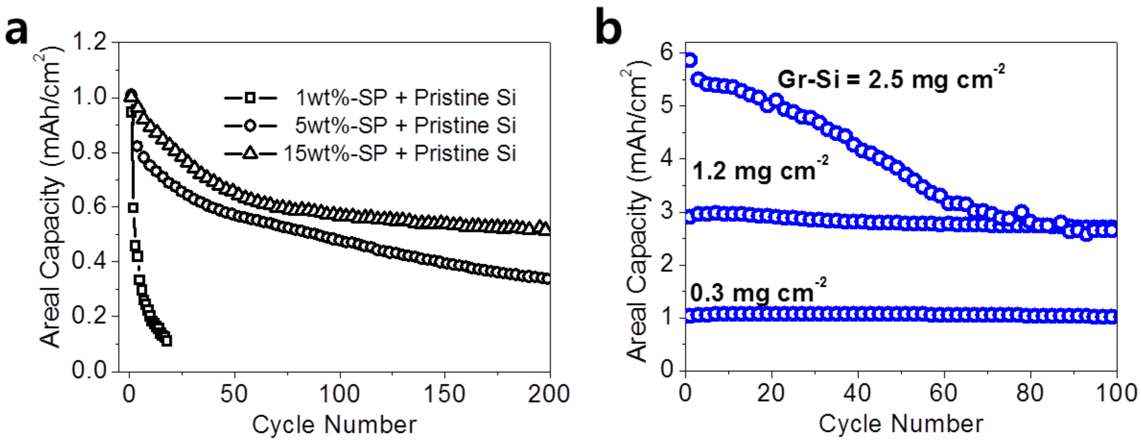

Supplementary Figure 8. Cycling performance. Areal capacity retentions of (**a**) pristine Si with various Super P contents and (**b**) 5wt%-Gr-Si with various areal loadings. The binder content was 20 wt%. All the electrodes were cycled at 0.5C for charge and discharge in each cycle. The mass loadings of Si + coated graphene for the different areal capacities are also denoted.

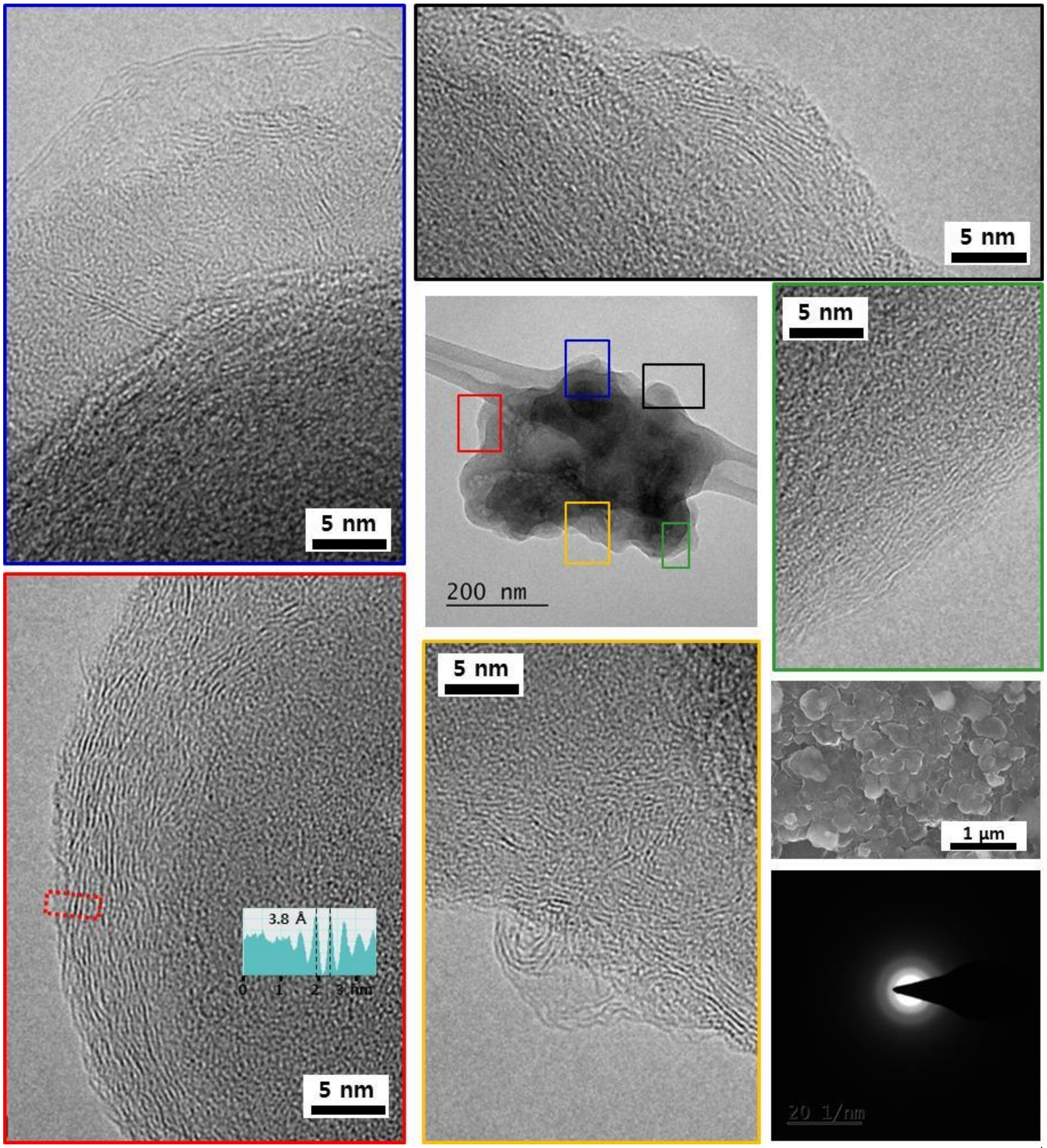

Supplementary Figure 9. *Ex-situ* TEM and SEM analysis of 5wt%-Gr-Si after the 1st lithiation. TEM and SAED images of 5wt%-Gr-Si NPs after the 1st lithiation. Close up HR-TEM images from the color boxes of the center TEM image clearly show graphene layers on Si surface.

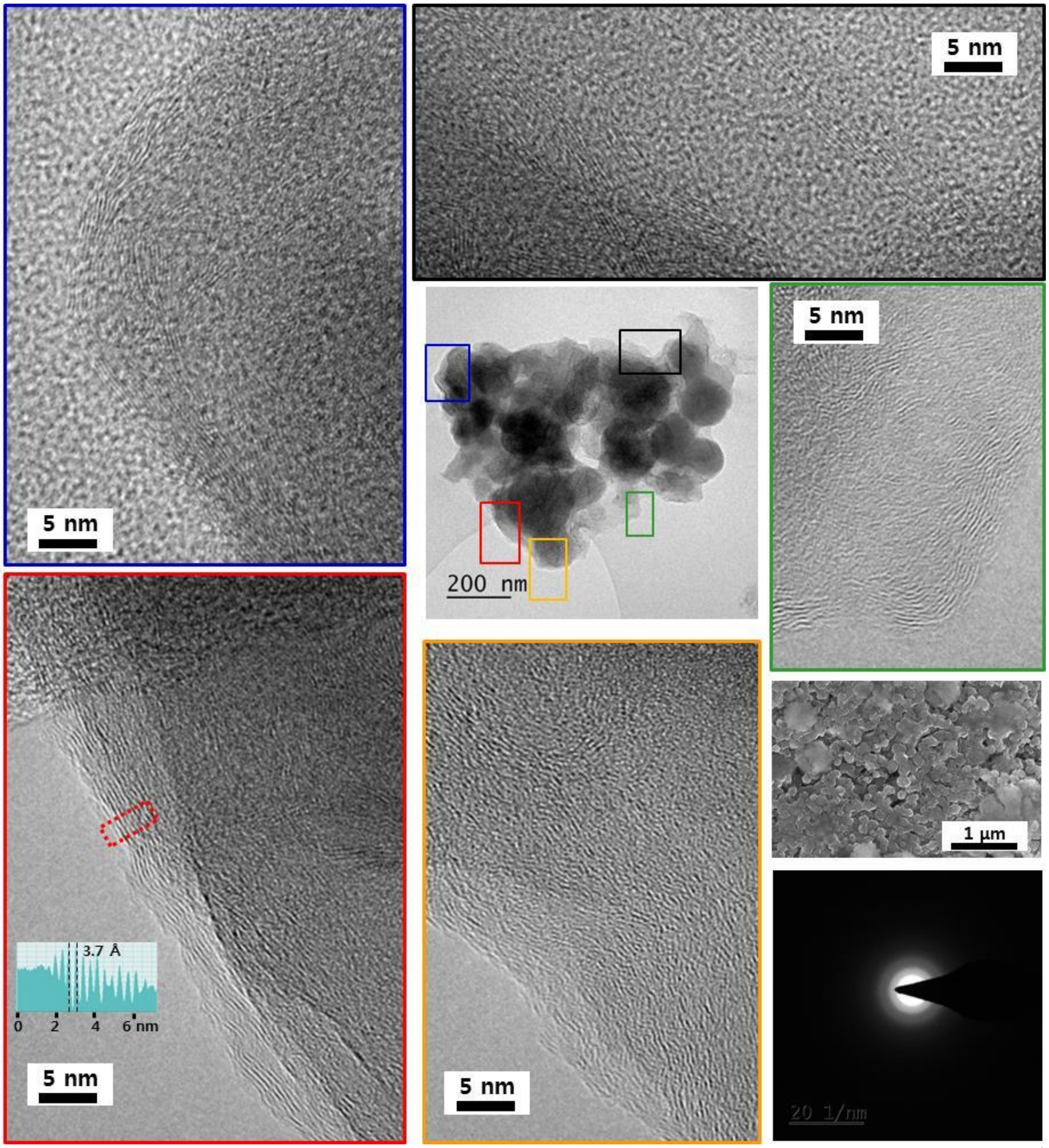

Supplementary Figure 10. The same analysis as Supplementary Fig. 9 but after the 1st delithiation.

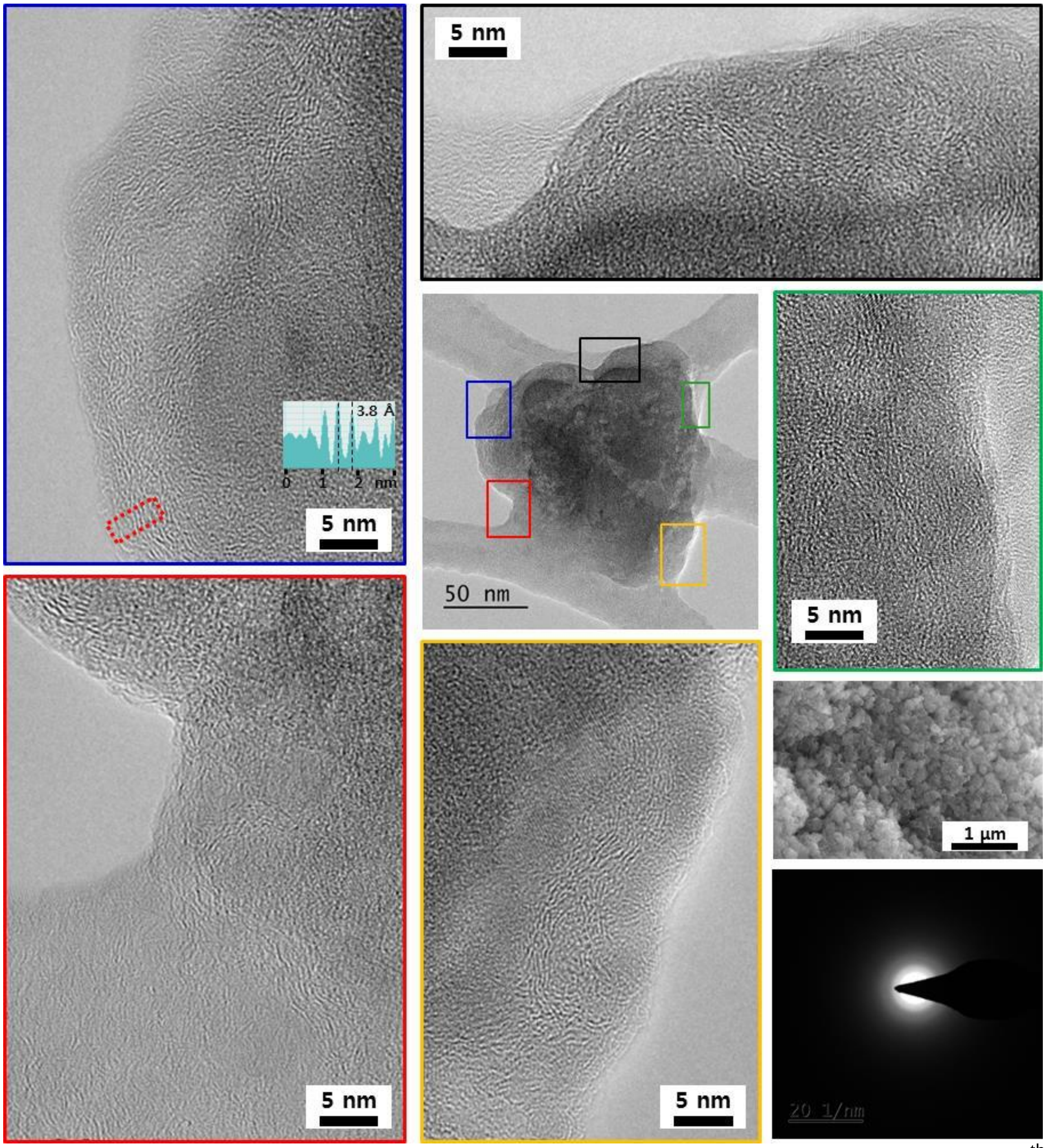

Supplementary Figure 11. The same analysis as Supplementary Fig. 9 but after the 200th delithiation.

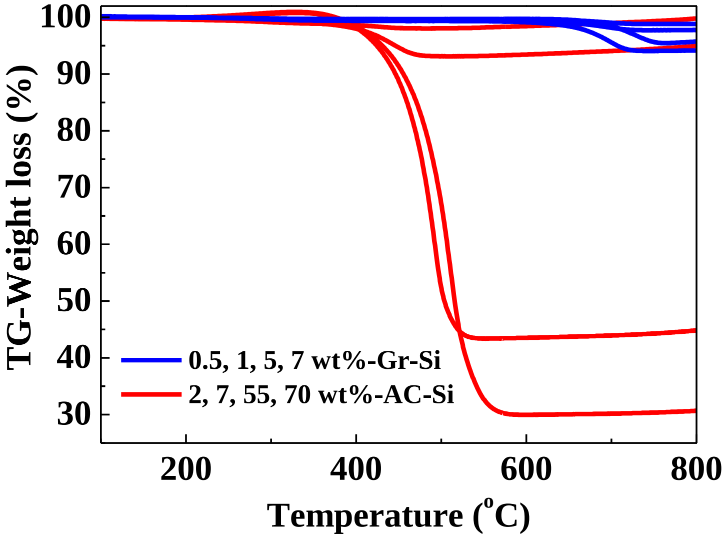

Supplementary Figure 12. Carbon content measurements for Gr-Si and AC-Si. TGA curves of both samples measured at a heating rate of 10 ^{o}C min^{-1} under air**.**
